# TXNDC9 promotes hepatocellular carcinoma progression by positive regulation of MYC-mediated transcriptional network

**DOI:** 10.1038/s41419-018-1150-4

**Published:** 2018-10-31

**Authors:** Dawei Chen, Jixue Zou, Zhenguo Zhao, Xiaodong Tang, Zhicheng Deng, Jingchao Jia, Shuanghai Liu

**Affiliations:** 1 Department of Hepatopancreatobiliary Surgery, Jiangyin People’s Hospital, School of Medicine, Southeast University, No. 163, Shoushan Road, Jiangyin, 214400 Jiangsu Province China; 2 Liver Cancer Institute, Zhongshan Hospital, Fudan University, No. 180, Fenglin Road, Shanghai, 200032 China

## Abstract

The thioredoxin domain containing proteins are a group of proteins involved in redox regulation and have been recently reported to be associated with tumor progression. However, the role of thioredoxin proteins in hepatocellular carcinoma (HCC) remains largely unknown. Here in our study, we demonstrated that thioredoxin domain containing protein 9 (TXNDC9) was over-expressed in HCC and promoted HCC progression. We found that TXNDC9 expression was amplified in HCC tissues and associated with an advanced grade of HCC. And, we demonstrated that overexpression of TXNDC9 was correlated with poor prognosis of HCC. Furthermore, by using CRISPR-Cas9 mediated TXNDC9 knockout and RNA-seq analysis, we found that TXNDC9 accelerated HCC proliferation regulation. Moreover, we demonstrated that TXNDC9 directly interacted with MYC and knockout/knockdown of TXNDC9 decreased the protein levels of MYC and inhibited MYC-mediated transcriptional activation of its targets. Besides, we identified that TXNDC9 was trans-activated by FOXA1, JUND, and FOSL2 in HCC. Taken together, our study unveiled an oncogenic role of TXNDC9 in HCC and provided a mechanistic insight into the TXNDC9 mediated gene regulation network during HCC development.

## Introduction

Hepatocellular carcinoma (HCC) is an increasingly serious health problem, with over 700,000 new cases every year worldwide^1–3^. HCC is characterized by a high and increasing incidence, a late diagnosis when curative-intent treatments are not feasible, a low resectability rate, a high recurrence after a curative-intent surgery, a poor response to medical treatments, and finally a grave prognosis^4^. The mortality rate due to HCC in most countries is nearly equal to the incidence rate, indicating the lack of effective therapies at diagnosis^5^. Hence, it is critical to explore novel biomarkers for HCC diagnosis, prediction of patient and treatment outcomes, and individualization of targeted therapies^6,7^.

Thioredoxin (TRX) domain containing proteins are a group of redox proteins that catalyze the reversible oxidation of cysteine thiol to disulfide^8,9^. This superfamily of proteins has roles in various functions in the regulation of redox homeostasis, oxidative stress, disulfide-containing proteins, and transcription^10,11^. Emerging evidence on cancer development has shown that the thioredoxin system contributed greatly to tumorigenesis and that aggressive tumors expressed high levels of thioredoxin^12–14^. Thioredoxin domain containing 9 (TXNDC9), also known as phosducin-like protein 3 (PHLP3), is a distinct member of thioredoxins, which have ATP binding protein activity. It modulates the ATPase activity of chaperonin TCP1 complex, a key complex for protein folding, and diminishes actin and tubulin folding^15^. Interestingly, recent studies have demonstrated that TXNDC9 was overexpressed in colorectal cancer, indicating a potential oncogenic function of TXNDC9 in malignant diseases^16^. However, the role of TXNDC9 in HCC and the mechanism by which TXNDC9 may exert an oncogenic function remains largely unknown.

Here, we demonstrated that TXNDC9 was highly expressed in HCC and that the high expression of TXNDC9 was a poor prognosis factor of HCC. The knockout/knockdown of TXNDC9 resulted in a significant inhibition of the growth of HCC cells. Moreover, we observed that TXNDC9 directly interacted with MYC and stabilized the MYC protein, and the knockout of TXNDC9 impaired the binding of MYC to chromatin. Finally, we found that FOXA1, JUND, and FOSL2 bound at the promoter of TXNDC9 and led to TXNDC9 overexpression in HCC.

## Materials and methods

### Enrolled patients

A total of 208 patients with HCC who had undergone surgical resection at Jiangyin People’s Hospital, School of Medicine, Southeast University from January 2010 to December 2014 were enrolled in this study. This study included 176 males and 32 females, with a mean age of 50.9 years old (from 25 to 75 years old). The follow-up period in this study ranged from 2 to 86 months after surgery. This research was performed in accordance with the ethical standards of Jiangyin People’s Hospital, School of Medicine, Southeast University and the Declaration of Helsinki and its amendments. Written consents were acquired from all participants.

### Tissue microarray (TMA) construction and immunohistochemistry

TMAs were constructed using HCC and paired adjacent noncancerous liver tissue specimens from each patient. The slides were immunolabeled by a primary rabbit monoclonal antibody against human TXNDC9 (1:100, Abcam, Cambridge, UK). The staining intensity for TXNDC9 was graded as 0 (negative), 1 (weak), or 2 (strong). The staining extent was graded as 0 (0%), 1 (1–25%), 2 (26–50%), 3 (51–75%), or 4 (76–100%). Specimens were classified into negative (0–1), weakly positive (2–4), or strongly positive (5–6), based on the sum of the staining intensity and staining extent scores. Weakly and strongly positive were considered as positive. The slides were analyzed by two independent researchers.

### RNA extraction and quantitative real-time polymerase chain reaction (qPCR)

Total RNA of frozen tissues was extracted using an All-Prep DNA/RNA Mini kit (Qiagen, Hilden, Germany). cDNA was reverse transcribed with the A3500 RT-PCR System (Promega Corporation, Madison, WI, USA) from 1 μg of total RNA. The relative TXNDC9 mRNA levels were detected by qPCR with Mastercycler^®^ ep realplex (Eppendorf, Hamburg, Germany) using an iQ^TM^ SYBR^®^ Green Supermix kit (Bio-Rad, Hercules, CA, USA). The mRNA levels were normalized to those of β-Actin. The following specific primers for qPCR were used: TXNDC9-F: 5′- CTGCTTCAGACTACCAAACTGG-3′, TXNDC9-R: 5′- CTCTGTAGAAATGGCAAACCACA-3′; SRM-F: 5′-GTGGTGGCCTATGCCTACTG-3′, SRM-R: 5′-CTCCTGGAAGTTCGTGCTCG-3′; DUSP2-F: 5′-GGGCTCCTGTCTACGACCA-3′, DUSP2-R: 5′-GCAGGTCTGACGAGTGACTG-3′; WDR74-F: 5′-CCTGGGGTGTGTAGGATGC-3′, WDR74-R: 5′-CAAGTCCAGCCAGTCATTCCG-3′; PES1-F: 5′-GGCCACCAACTACATCACCC-3′, PES1-R: 5′-AGAATGCACAGCCGCCTAAA-3′; β-Actin-F: 5′- CATGTACGTTGCTATCCAGGC-3′, β-Actin-R: 5′-CTCCTTAATGTCACGCACGAT-3′. The relative mRNA expression levels were evaluated using the 2^−ΔΔCT^ comparative method.

### Western blot

Total protein, cytoplasmic and nuclear protein were isolated and quantified by a bicinchoninic acid protein assay kit (Beyotime Biotechnology Co., Jiangsu, China). Then, aliquots of proteins were separated on a 10% sodium dodecyl sulfate-polyacrylamide gel electrophoresis and transferred to polyvinylidine difluoride filter membranes (Millipore, Bedford, MA, USA). The membranes were blocked with 5% nonfat milk and probed by a primary rabbit monoclonal antibody against human TXNDC9 (1:1000, Abcam, Cambridge, UK), GAPDH (1:5000, CST, Danvers, MA, USA) or rabbit polyclonal antibody MYC (1:1000, Santa Cruz Biotechnology, CA, USA). Following incubation with a horseradish peroxidase-conjugated secondary antibody (1:3000, Santa Cruz Biotechnology, CA, USA), blots were visualized by chemiluminescence (Millipore, Bedford, MA, USA). GAPDH was used as the loading control.

### CRISPR-Cas9 mediated TXNDC9 knockout

The HepG2 and Hep3B cell lines were purchased from the American Type Culture Collection (ATCC) and cultured in DMEM containing 10% of fetal bovine serum. The CRISPR-Cas9 system was used to create the TXNDC9 knockout. The sgRNA sequences, targeting the first exon of TXNDC9, were inserted to the lentiCRISPRv2 plasmid (Addgene Plasmid #52961)^17^. The sgRNA sequences are listed below. 5′-GTTGAGCTTTCCTTAGTGCC-3′ or 5′-TCTGAAGCAGCTGATGCTCC-3′. Lentivirus was produced as previously described^17^.

### RNA interference

siRNAs were purchased from the Genepharm co. ltd. siRNA primers are listed below. si-FOXA1-sense: 5′-GAGAGAAAAAAUCAACAGCdTdT-3′, si-FOXA1-antisense: 5′-GCUGUUGAUUUUUUCUCUCdTdT-3′; si-JUND-sense: 5′- CCCUCAAGAGUCAGAACACdTdT-3′, si-JUND-antisense: 5′- GUGUUCUGACUCUUGAGGGdTdT-3′; si-FOSL2-sense: 5′- GGAUUAUCCCGGGAACUUUdTdT-3′, si-FOSL2-antisense: 5′-AAAGUUCCCGGGAUAAUCCdTdT-3′. siRNAs were transfected into HepG2 cells using lipofectamine 2000 (ThermoFisher Scientific, MA, USA) according manufactures’ instructions. shRNA targeting TXNDC9 were constructed in plvx-sh2 vector with the following primer. shTXNDC9-F: 5′- GATCCGCGGTTCTTCTGACATTCTTAATTCAAGAGATTAAGAATGTCAGAAGAACCGTTTTTTG-3′, shTXNDC9-R: 5′- AATTCAAAAAACGGTTCTTCTGACATTCTTAATCTCTTGAATTAAGAATGTCAGAAGAACCGCG-3′.

### RNA-seq

RNA was extracted from TXNDC9 knockout or wild-type HepG2 cells using TRIzol Reagent (ThermoFisher Scientific, MA, USA) according to the manufacturer’s protocol. Polyadenylated RNA was isolated from 5 μg total RNA with the mRNA Capture Beads. An RNA-seq library was constructed with VAHTSTM mRNA-seq V2 Library Prep Kit for Illumina^®^ (Vazyme Biotech Co., Nanjing, China) according to the manufacturer’s protocol. Sequencing was performed using the Illumina Hiseq Xten platform.

### RNA-seq data processing

Reads were mapped to human genome (hg38) using TopHat2^18^ with a reference annotation from the Ensembl database. No-novel-juncs options were used to ignore de novo gene and transcript identification. A gene expression profile of each sample, as well as differentially expressed genes between wild-type and TXNDC9 knockout HepG2 cells, were identified using the Cuffdiff suite from Cufflinks^19^. Genes with two-fold changes between two groups, *q* value < 0.01 and average expression > 1 were identified as TXNDC9 regulated targets. Raw data and processed data were deposit at the GEO database with following links: https://www.ncbi.nlm.nih.gov/geo/query/acc.cgi?acc=GSE113400 with token key: gzmjeceqpnohler.

### Chromatin immunoprecipitation (ChIP)

ChIP experiments were performed with the ChIP-IT high sensitivity kit (Active motif, CA, USA) in TXNDC9 knockout and wild-type HepG2 cells according to the manufacture’s instruction. Antibodies against MYC (Santa Cruz Biotechnology, CA, USA) were used. ChIP-qPCR primers were used as list below. EIF3B- ChIP-q-F: GGTCAATGCTCCCATGAGTT; EIF3B-ChIP-q-R: AGGACTCGCATCCACAAGAC; EIF4A1-ChIP-q-F: GGGATGTGGATTGTTCATGG; EIF4A1-ChIP-q-R: GGGGAGATTGCACAACACTT; CDK4-ChIP-q-F: TGGGAAGGGACTGCACTTAC; CDK4-ChIP-q-R: GAGGGGGCCTCTCTAGCTT; Negative-F: TTAGACGGAGTCGGAGCTGT; Negative-R: TCTTCAGGTCGGGCATTATC.

### Transcription enrichment analysis

The ENCODE ChIP-seq Significance tool (http://encodeqt.simple-encode.org) was used for transcription enrichment analysis. In all, −2000 to 200 bp of the transcription start site of genes regulated by TXNDC9, identified by RNA-seq analysis, were used for transcription factor enrichment.

### Statistical analysis

Correlation of TXNDC9 expression with clinicopathologic characteristics was assessed by Pearson’s *χ*^2^ test. The overall survival (OS) was evaluated by the Kaplan–Meier method using the log-rank test. Independent risk factors for OS were investigated by Cox regression analysis. Statistical analysis was conducted with SPSS 21.0 (IBM, Armonk, NY, USA). All tests were two-sided, and a statistically significant difference was considered when *p* < 0.05.

## Results

### TXNDC9 expression is significantly upregulated in human HCC tissues

**Fig. 1:**
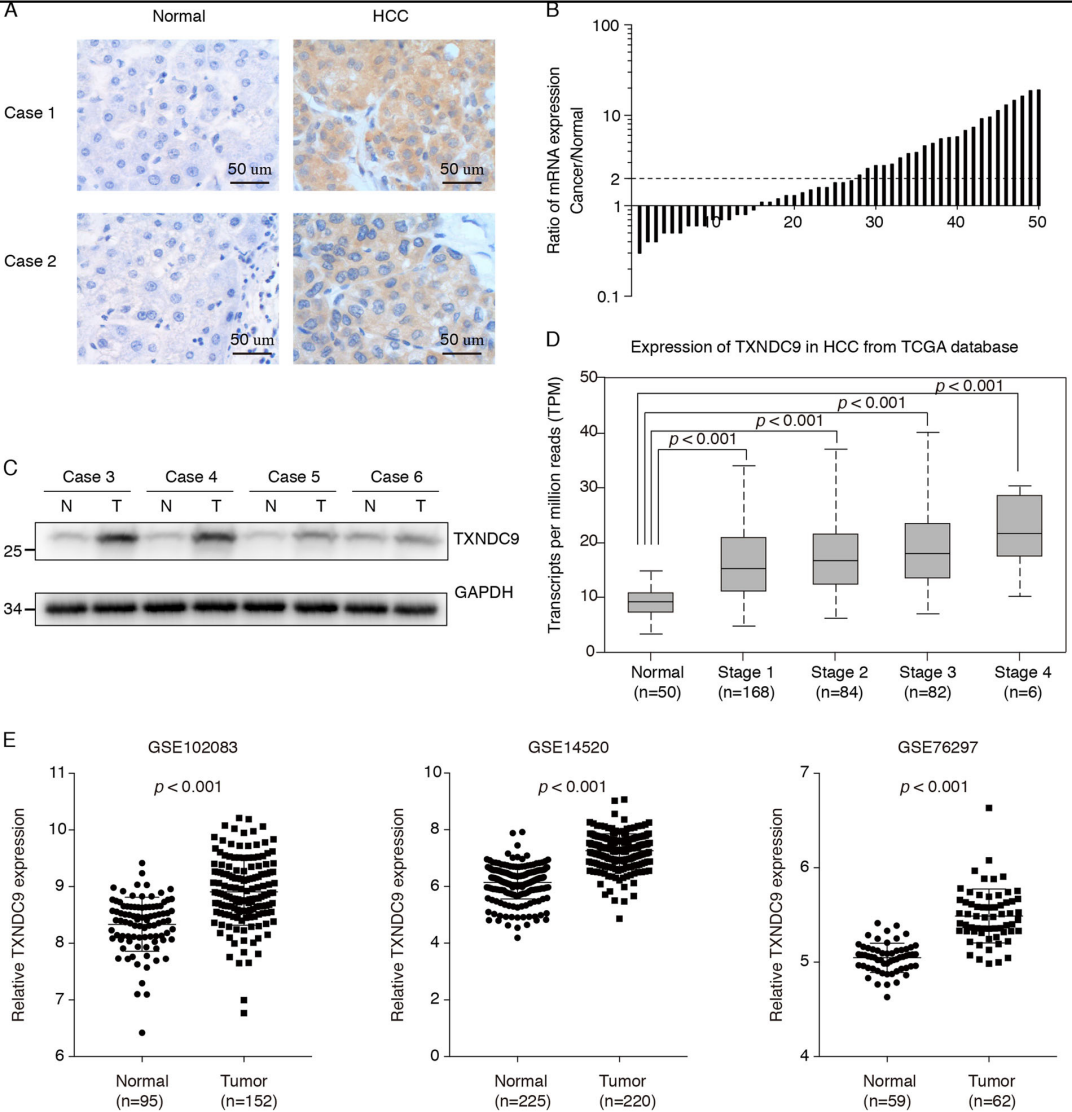
TXNDC9 was overexpressed in HCC. **a** Immunohistochemical staining of HCC tissues and paired normal liver tissues. Two representative cases are shown. **b** Waterfall plot of relative TXNDC9 mRNA expression of 50 HCC tissues and paired normal liver tissues measured using qPCR. **c** Western blot analysis of TXNDC9 protein expression in four representative HCC tissues and paired normal liver tissues. “N” represents normal liver tissue. “T” represents HCC tissue. **d** TXNDC9 gene expression levels were assessed from TCGA. Amplification of the TXNDC9 gene expression was associated with advanced disease stage. Error bars represent the mean ± SD. **e** TXNDC9 gene expression levels were assessed from GEO database. Error bars represent the mean ± SD

To determine the effect of TXNDC9 expression on HCC progression, we examined TXNDC9 expression in HCC tissues and paired adjacent noncancerous liver tissues. We performed immunohistochemical staining of TMAs containing 208 paired samples of HCC and normal liver tissues, and observed that the frequency of TXNDC9-positive staining in cancerous tissues (70.7%, 147/208) was statistically significantly higher than that observed in normal liver tissues (28.8%, 60/208) (*p* *<* 0.001, Fig. 1a). The mRNA expression level of TXNDC9 was investigated by qPCR in 50 paired HCC and normal liver tissues that were randomly selected from the above 208 cases. In agreement with the results from immunohistochemical staining, TXNDC9 mRNA levels in HCC tissues were significantly upregulated in 23 of 50 cases (46.0%, fold change ≥ 2, Fig. 1b). We next selected several pairs of these samples and validated TXNDC9 expression using western blot. Consistent with the results above, the western blot analysis demonstrated that HCC tissues had higher TXNDC9 protein expression compared with normal liver tissues (Fig. 1c).

To further confirm our finding that TXNDC9 is aberrantly overexpressed in HCC, we analyzed the expression of TXNDC9 in HCC and normal liver tissues using datasets from The Cancer Genome Atlas (TCGA) database and seven clinical cohorts from GEO database. In accordance with our findings, the expression of TXNDC9 in HCC tissues were significantly higher than those in normal liver tissues (Figs. 1d, e and Figure S1), suggesting an oncogenic role of TXNDC9 in HCC progression.

### Correlation of TXNDC9 expression with clinicopathologic characteristics

Because TXNDC9 expression was significantly increased in HCC, we sought to determine whether TXNDC9 expression according to the TMAs immunohistochemical staining results was associated with the clinicopathologic characteristics of HCC patients (Table 1). High TXNDC9 expression was positively correlated with tumor-node-metastasis (TNM) stage (*p* *=* 0.035). This was further supported by the TCGA datasets, as illustrated in Fig. 1d, patients with a more aggressive stage tend to express a higher level of TXNDC9. In addition, we also observed that positive TXNDC9 expression was significantly related to tumor size > 5 cm (*p* = 0.013) and poor tumor differentiation (*p* = 0.022). However, there was no significant correlation between TXNDC9 expression and gender, age, hepatitis B virus DNA, alpha-fetoprotein, cirrhosis, tumor number, or portal vein invasion (all *p* > 0.05). Thus, TXNDC9 is up-regulated in HCC with advanced grade and might serve as an oncogene during HCC development and progression.

**Table 1:** Correlations between TXNDC9 expression and clinicopathologic variables

Variables	TXNDC9 expression in HCC		*p*
	Negative (*n* = 61, %)	Positive (*n* = 147, %)	
Gender			0.871
Male	52 (85.2%)	124 (84.4%)	
Female	9 (14.8%)	23 (15.6%)	
Age			0.403
≤50 years	35 (57.4%)	75 (51.0%)	
>50 years	26 (42.6%)	72 (49.0%)	
Hepatitis B virus DNA			0.822
≤5 log10 copies/mL	51 (83.6%)	121 (82.3%)	
>5 log10 copies/mL	10 (16.4%)	26 (17.7%)	
AFP			0.109
≤200 ng/mL	34 (55.7%)	64 (43.5%)	
>200 ng/mL	27 (44.3%)	83 (56.5%)	
Cirrhosis			0.725
No	9 (14.8%)	19 (12.9%)	
Yes	52 (85.2%)	128 (87.1%)	
Tumor number			0.188
Single	44 (72.1%)	92 (62.6%)	
Multiple	17 (27.9%)	55 (37.4%)	
Tumor size			0.013
≤5 cm	32 (52.5%)	50 (34.0%)	
>5 cm	29 (47.5%)	97 (66.0%)	
Portal vein invasion			0.461
No	52 (85.2%)	119 (81.0%)	
Yes	9 (14.8%)	28 (19.0%)	
Tumor differentiation			0.022
Well and moderate	44 (72.1%)	81(55.1%)	
Poor	17 (27.9%)	66 (44.9%)	
TNM stage			0.035
I–II	38 (62.3%)	68 (46.3%)	
III–IV	23 (37.7%)	79 (53.7%)	

### TXNDC9 overexpression is an independent prognostic indicator of a poor HCC survival

**Fig. 2:**
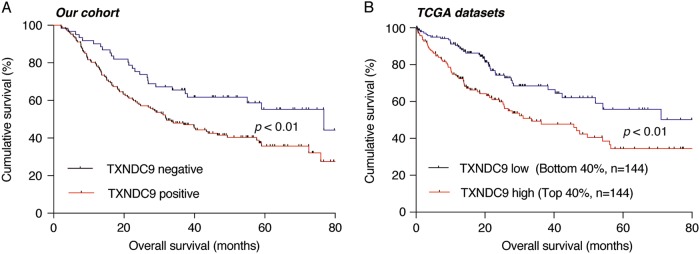
TXNDC9 overexpression was correlated with poor prognosis in HCC patients. **a** Kaplan–Meier plots of overall survival based on the TXNDC9 expression in HCC tissues of our cohort with the log-rank test. **b** Kaplan–Meier plots of overall survival based on TXNDC9 expression in HCC tissues of the TCGA cohort. The top 40% of TXNDC9 expression were identified as TXNDC9 high-expression group, while the bottom 40% of TXNDC9 expression were identified as TXNDC9 low-expression group, and log-rank analysis was used to test for significance

To further investigate whether over-expressed TXNDC9 may contribute to HCC prognosis, we performed a Kaplan–Meier and Cox regression analysis according to the TMAs immunohistochemical staining results. The difference of OS between patients with TXNDC9-positive tumors and those with TXNDC9-negative tumors was statistically significant by the Kaplan–Meier method. The 5-year OS rate of patients with TXNDC9-positive tumors was remarkably lower than that of patients with TXNDC9-negative tumors (35.7 vs. 55.3%, *p* < 0.010, Fig. 2a). In addition, we also analyzed the effect of TXNDC9 overexpression on survival using the TCGA datasets. As demonstrated in Fig. 2b, the TXNDC9 overexpression was shown to be a poor prognostic factor in HCC (*p* < 0.010). Furthermore, the association between 11 clinicopathologic characteristics and survival rate of HCC patients after surgical resection was investigated by Cox regression analysis. Univariate Cox regression analysis indicated that multiple tumors (*p* = 0.014), tumor size > 5 cm (*p* = 0.008), portal vein invasion (*p* = 0.007), poor tumor differentiation (*p* = 0.018), TNM stage III–IV (*p* = 0.001), and positive TXNDC9 expression (*p* = 0.011) were predictors for a lower OS (Table 2). These characteristics of HCC patients, which were associated with survival in the univariate analysis, were further evaluated in a multivariate Cox model. Multivariate analysis demonstrated that TNM stage III–IV (HR, 1.756; 95% CI, 1.205–2.560; *p* = 0.003) and positive TXNDC9 expression (HR, 1.629; 95% CI, 1.047–2.534; *p* = 0.030) may predict a lower OS independently (Table 2).

**Table 2:** Cox regression analysis of the risk factors for overall survival rate

	Univariate analysis		Multivariate analysis	
	HR (95% CI)	*p*	HR (95% CI)	*p*
Gender		0.667		–
Male				
Female	0.890 (0.525, 1.511)		–	
Age		0.871		–
≤50 years				
>50 years	0.970 (0.672, 1.400)		–	
Hepatitis B virus DNA		0.550		–
≤5 log10 copies/mL				
>5 log10 copies/mL	1.155 (0.720, 1.855)		–	
AFP		0.147		–
≤200 ng/mL				
>200 ng/mL	1.315 (0.908, 1.903)		–	
Cirrhosis		0.486		–
No				
Yes	1.214 (0.704, 2.094)		–	
Tumor number		0.014		0.369
Single				
Multiple	1.591 (1.097, 2.306)		–	
Tumor size		0.008		0.265
≤5 cm				
> 5 cm	1.701 (1.149, 2.519)		–	
Portal vein invasion		0.007		0.238
No				
Yes	1.799 (1.174, 2.758)		–	
Tumor differentiation		0.018		0.056
Well and moderate				
Poor	1.559 (1.079, 2.251)		–	
TNM stage		0.001		0.003
I–II				
III–IV	1.860 (1.279, 2.703)		1.756 (1.205, 2.560)	
TXNDC9 expression		0.011		0.030
Negative				
Positive	1.766 (1.140, 2.737)		1.629 (1.047, 2.534)	

### Knockout TXNDC9 significantly inhibit HepG2 cell growth

**Fig. 3:**
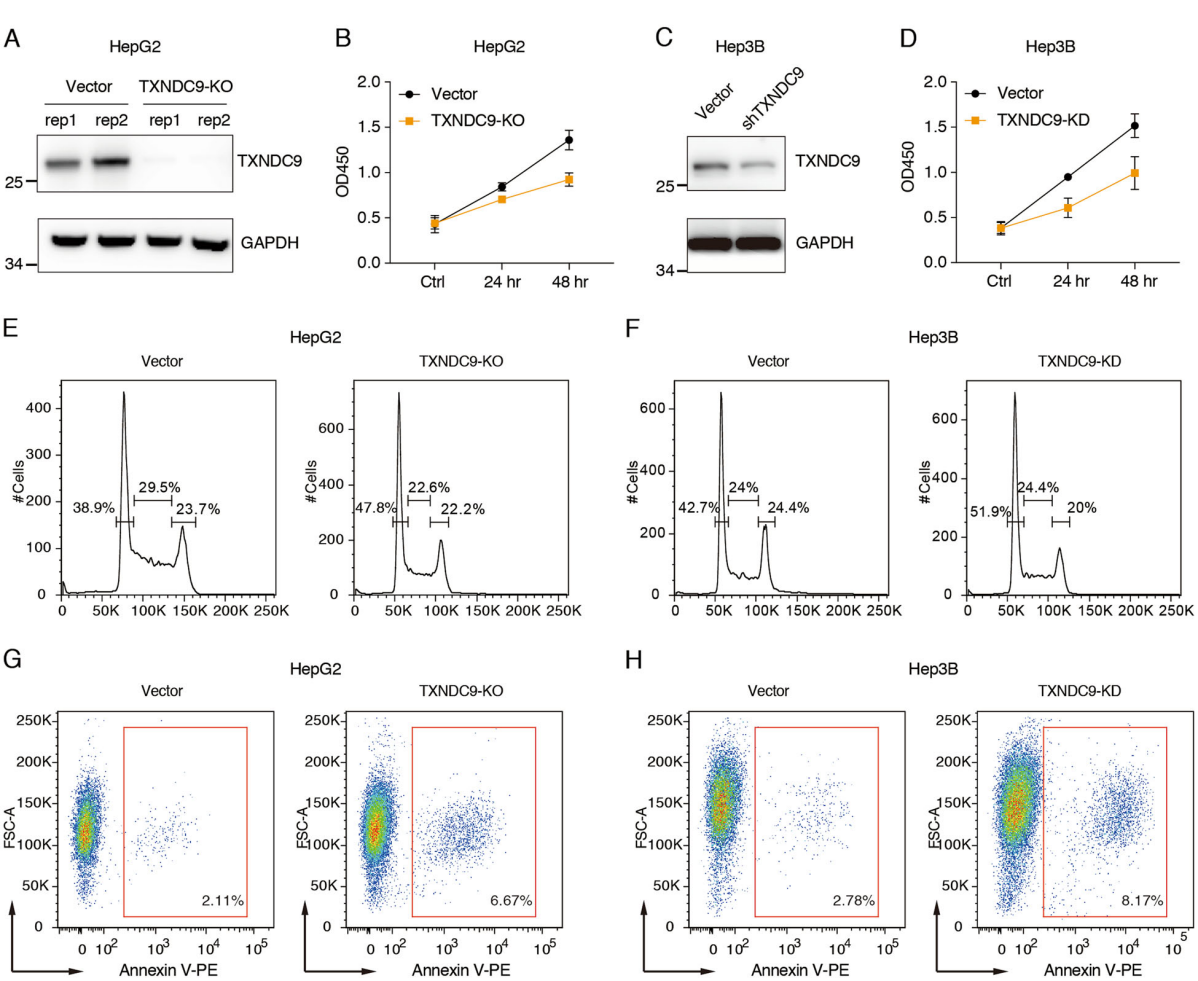
Knockout/knockdown of TXNDC9 impaired the growth of HCC cells. **a** Western blot analysis of TXNDC9 expression in whole cell lysates. HepG2 cells were harvested after FACS sorting of cells positively infected with Cas9 as well as sgRNA against TXNDC9 or vector alone. **b** Knockout of TXNDC9 inhibited the cell proliferation of HepG2 cells. Cell proliferation rates were measured by CCK8 assay. The cell densities were measured at 450 nm OD. Error bars represent the mean ± SD from three independent experiments. **c** Western blot analysis of TXNDC9 expression in Hep3B cells with TXNDC9 knockdown. Hep3B cells were harvested after FACS sorting of cells positively infected with shRNA targeting TXNDC9 or vector alone. **d** Knockdown of TXNDC9 inhibited cell proliferation of Hep3B cells. **e**, **f** Knockout/knockdown of TXNDC9 induced G0/G1 phase arrest of HepG2 or Hep3B cells. The representative DNA content histograms are shown. **g**, **h** Knockout/knockdown of TXNDC9 induced apoptosis of HCC cells. The Annexin-V positive cells were gated as apoptotic cells

The correlation of TXNDC9 overexpression and HCC progression suggested an oncogenic role of TXNDC9 in HCC. To investigate the exact role of TXNDC9 in HCC cells, we first knocked out the expression of TXNDC9 in a human HCC cell line (HepG2). As shown in Fig. 3a, the expression of TXNDC9 was significantly reduced in cells co-transfected with CRISPR-Cas9 and sgRNA against the TXNDC9. We next examined whether TXNDC9 knockout affected the cell growth of HepG2 cells. As shown in Fig. 3b, cell proliferation was significantly inhibited by TXNDC9 knockout, indicating that TXNDC9 overexpression is required for HCC proliferation. We further validated this observations in Hep3B cell lines using lentiviral mediated knockdown of TXNDC9. As shown in Fig. 3c, d, knockdown of TXNDC9 also inhibited the cell proliferation of Hep3B cells. Since cell growth was affected, we next sought to determine whether cell cycle was altered by TXNDC9 knockout. As shown in Fig. 3e, f, cell cycle arrest was also observed in TXNDC9 knockout/knockdown cells. Taken together, these results indicate that TXNDC9 knockout/knockdown could indeed affect HCC progression via cell growth inhibition. In addition, we also determined cell apoptosis upon TXNDC9 knockout/knockdown. As shown in Fig. 3g, h, decreased TXNDC9 expression also induced a moderate apoptosis in HCC cells.

### TXNDC9 involved in multiple biological process in HCC

**Fig. 4:**
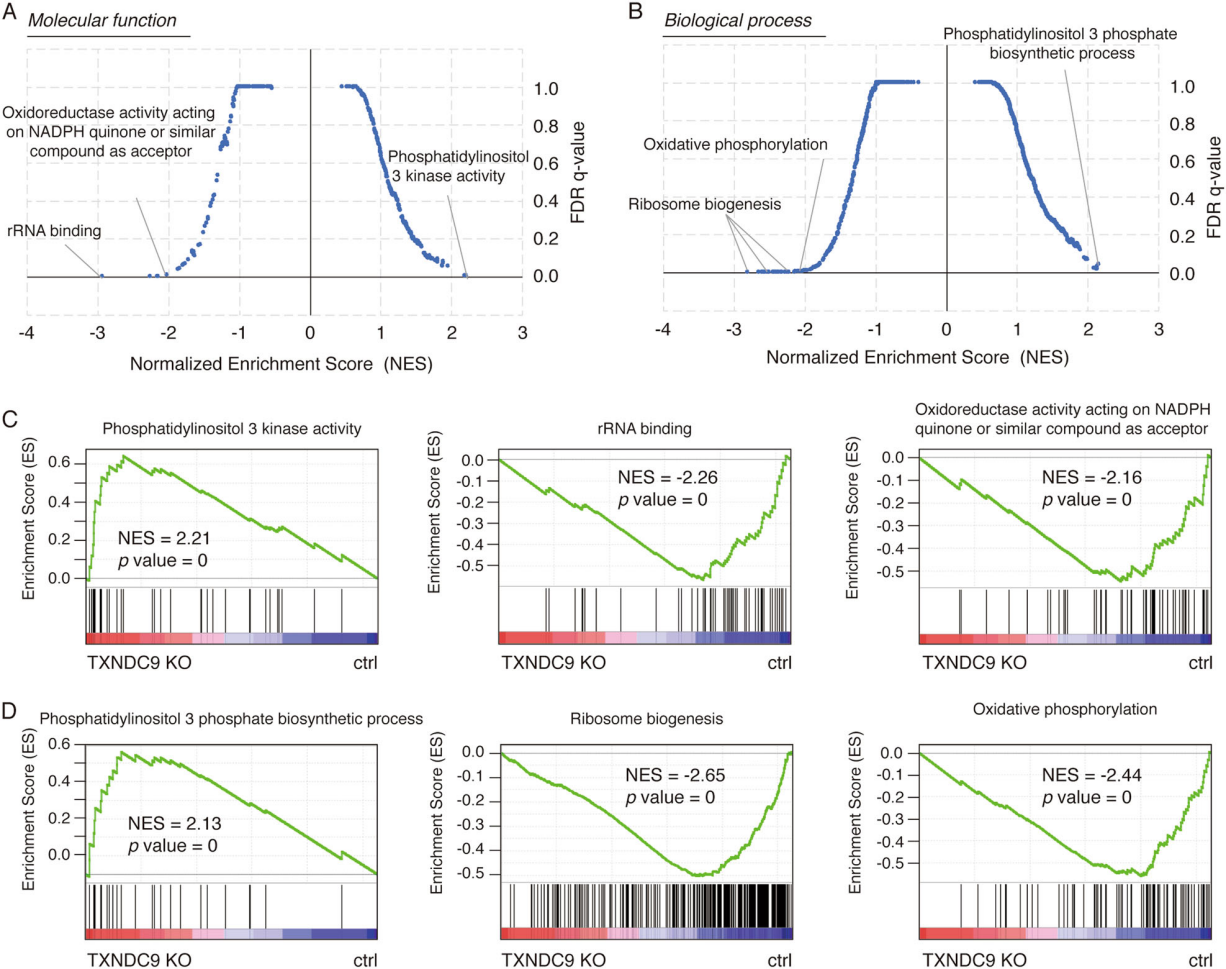
Gene ontology enrichment of genes regulated by TXNDC9. **a** Enrichment of molecular function gene sets in TXNDC9 knockout. Gene set enrichment analysis were performed using the GSEAP reranked tools with the molecular function gene sets from MSigDB database, Enrichment score and relative *q*-value were plotted. **b** Enrichment of biological process gene sets in TXNDC9 knockout. Gene set enrichment analysis were performed using the GSEAP reranked tools with the biological process gene sets from MSigDB database, Enrichment score and relative *q*-value were plotted. **c** Representative enrichment map of molecular function gene sets in TXNDC9 knockout. **d** Representative enrichment map of biological process gene sets in TXNDC9 knockout

To determine how TXNDC9 affected the progression of HCC, we performed RNA-seq analysis in TXNDC9 knockout and wild-type HepG2 cells. We identified 1758 genes were up-regulated while 1783 genes were down-regulated upon TXNDC9 knockout (Table S1). We then performed gene set enrichment analysis using the gene ontology gene sets including molecular functions and biological processes. For molecular functions analysis, as shown in Fig. 4a, c, genes involved in phosphatidylinositol 3 kinase activity were associated with genes up-regulated upon TXNDC9 knockout while genes involved in rRNA binding and oxidoreductase activity were associated with genes down-regulated upon TXNDC9 knockout (Table S2). For biological processes analysis, genes involved in phosphatidylinositol 3 phosphate biosynthetic process were associated with genes up-regulated upon TXNDC9 knockout while genes involved in ribosome biogenesis and oxidative phosphorylation were associated with genes down-regulated upon TXNDC9 knockout (Fig. 4b, d and Table S3).

### TXNDC9 is involved in MYC-mediated transcriptional regulation

**Fig. 5:**
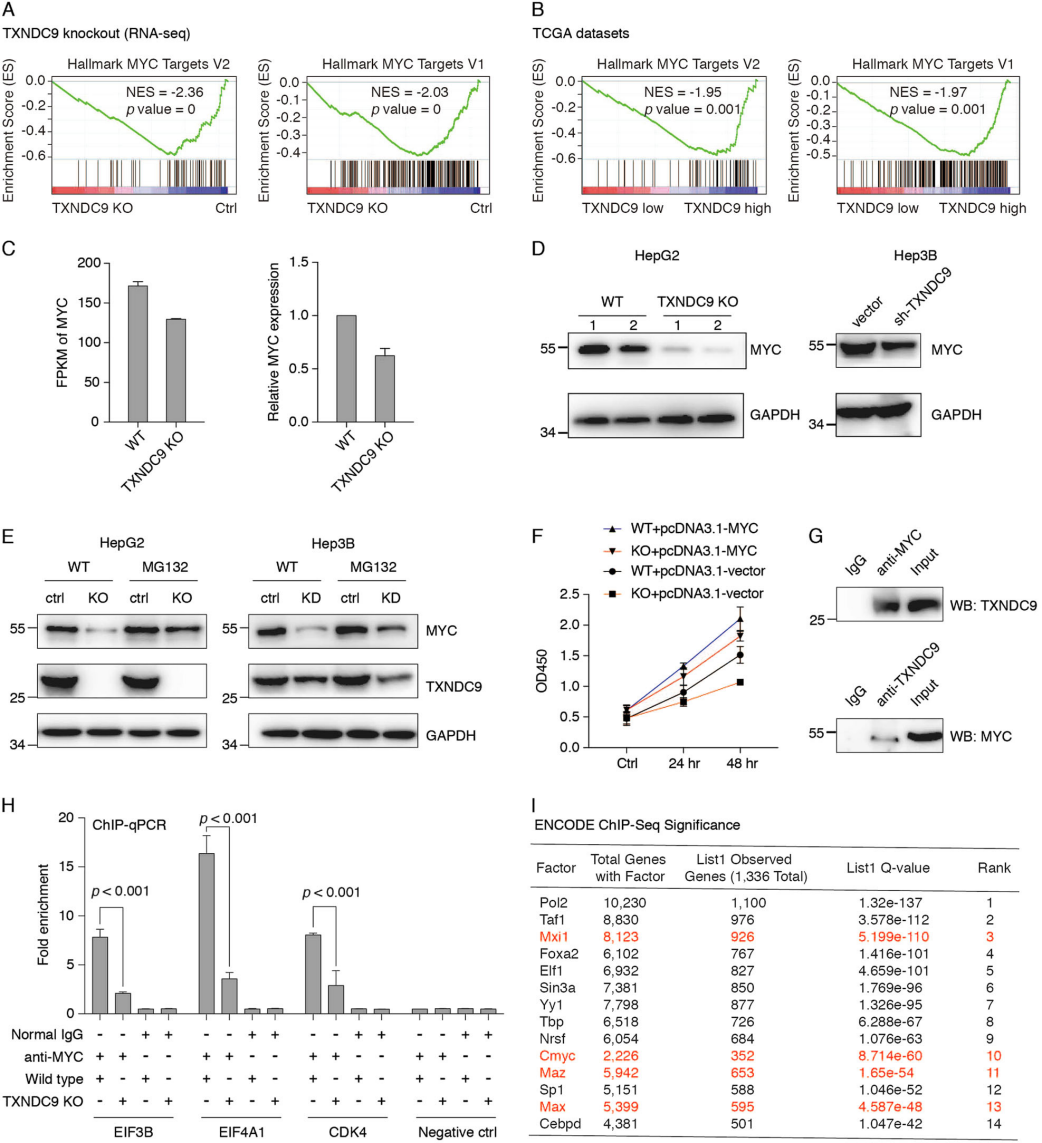
TXNDC9 cooperate with MYC in gene regulation. **a** MYC targets were associated with genes down-regulated upon TXNDC9 knockout. Gene set enrichment analysis were performed using the GSEAP rerank tools from the GSEA suite. Rank list were generated by log2 fold change of gene expression of wild-type HepG2 cells comparing to TXNDC9 knockout HepG2 cells. **b** GSEA analysis using the TCGA datasets. Rank list were generated by log2 fold change of average gene expression in patients with TXNDC9 high expression comparing to patients with TXNDC9 low expression. **c** TXNDC9 knockout induced modest downregulation of MYC mRNA. mRNA expression of MYC in TXNDC9 knockout and wild-type HepG2 cells were detected by RNA-seq and RT-qPCR. Error bars represent the mean ± SD from three independent experiments. **d** Knockout/knockdown of TXNDC9 decreased the protein level of MYC in HepG2 or Hep3B cells. Whole cell lysate of TXNDC9 knockout/knockdown and wild-type HepG2 or Hep3B cells were extracted and the protein level of MYC and GAPDH were detected. **e** Down-regulation of MYC upon TXNDC9 knockout/knockdown was proteasome dependent. Western blot was performed in TXNDC9 knockout/knockdown or wild-type HepG2 or Hep3B cells with or without MG132 treatment. The protein levels of MYC and TXNDC9 were determined, GAPDH were used as internal control. **f** Restoration of MYC abolished the proliferation inhibition induced by TXNDC9 knockout. Cell proliferation were determined by CCK8. **g** MYC directly interacted with TXNDC9 in the cytoplasm of HepG2 cells. Co-immunoprecipitation experiments were performed using cytoplasmic lysate of HepG2 cells. **h** Knockout of TXNDC9 decreased the MYC binding on its target genes. ChIP-qPCR were performed in TXNDC9 knockout and wild-type HepG2 cells with antibodies against MYC. Error bars represent the mean ± SD from three independent experiments. **i** Transcription factor binding sites enriched in the promoter of TXNDC9 regulated genes with the ENCODE ChIP-seq significance tool

To determine the function associated with TXNDC9 regulation, we performed gene set enrichment analysis using the 50 hallmark gene sets in the GSEA database. Interestingly, we found that MYC targets were significantly enriched at genes down-regulated upon TXNDC9 knockout (Fig. 5a). To validate this observation in primary samples, we ranked gene expression according to the expression level of TXNDC9 using the HCC expression samples from TCGA database, and performed gene set enrichment analysis using the hallmark gene sets. Indeed, we also observed that the MYC targets were significantly enriched in samples with TXNDC9 overexpression (Fig. 5b). Together, these results suggest that TXNDC9 might be required for the transcriptional activation of MYC. To determine this, we first examined whether expression of MYC mRNA was dependent on TXNDC9, however, we found that mRNA expression of MYC and its partner MXI1, MAX, MXD1 and MAZ were only slightly affected by TXNDC9 knockout (Fig. 5C and Figure S2) using both RNA-seq and RT-qPCR results. This suggests that TXNDC9 does not regulate MYC mRNA expression in HCC. We next investigated the protein level of MYC by western blot, and we found that it was significantly reduced by TXNDC9 knockout/knockdown (Fig. 5d). To test if the decreased MYC protein upon TXNDC9 knockout/knockdown was dependent on proteasome pathway, we treated the TXNDC9 knockout/knockdown cells with MG132, a typical proteasome inhibitor, and we found that the protein levels of MYC and the expression of down-regulated genes with TXNDC9 knockdown/knockout were significantly restored upon MG132 treatment (Fig. 5e and Figure S3).

Furthermore, to evaluate the functional importance of MYC in TXNDC9 mediated regulation, we restored the expression of MYC and determined the cell proliferation and apoptosis upon TXNDC9 knockout. As shown in Fig. 5f and Figure S4, overexpressed MYC significantly disrupt the cell growth inhibition and apoptosis induced by TXNDC9 knockout, suggesting that the major effects of TXNDC9 knockout were achieved via its association with MYC.

To further determine the role of TXNDC9 in gene regulation, we analyzed the distribution of TXNDC9 in HCC cells. As shown in Figure S5, TXNDC9 mainly expressed in cytoplasm, suggesting it might not be directly involved in transcriptional regulation. But the importance of MYC in TXNDC9-mediated regulation and the localization of TXNDC9 (Figure S5) prompt us to further evaluate the association between MYC and TXNDC9. We next performed co-IP experiments using antibodies against MYC and TXNDC9. As shown in Fig. 5g, the MYC directly interacted with TXNDC9 in the cytoplasmic lysate of HepG2 cells, suggesting that TXNDC9 might regulate the posttranslational modification of MYC or involved in the stabilization of MYC.

To better understand the role of TXNDC9 in the MYC-mediated transcriptional program, we performed ChIP-qPCR using antibodies against MYC in TXNDC9 knockout and wild-type HepG2 cells. Interestingly, we found the binding of MYC on its target genes was significantly reduced in the TXNDC9 knockout cells (Fig. 5h), indicating that TXNDC9 is essential for MYC-mediated transcriptional regulation.

Next, we performed ChIP-seq enrichment analysis of the TXNDC9 regulated genes using the ChIP-seq data from the Encyclopedia of DNA Elements (ENCODE) project to validate the finding that TXNDC9 regulates MYC targets. Indeed, we found that the promoter of genes down-regulated upon TXNDC9 knockout were significantly enriched at genome-wide binding sites of c-MYC as well as its partner MXI1, MAZ, and MAX (Fig. 5i). This further supports the theory that TXNDC9 is associated with the MYC-mediated transcriptional network.

### TXNDC9 overexpression is driven by FOXA1, JUND, and FOSL2

The mechanism by which TXNDC9 is overexpressed in HCC remains unanswered. To address this issue, we first investigated DNA methylation of the TXNDC9 promoter using the DNA methylation array results from TCGA database (Figure S6). In HCC and its adjacent normal tissues, the DNA methylation of TXNDC9 appeared to be similar and rare. This suggests the CpG island for TXNDC9 is not methylated and also is not the cause of TXNDC9 overexpression as compared to normal control tissues.

**Fig. 6:**
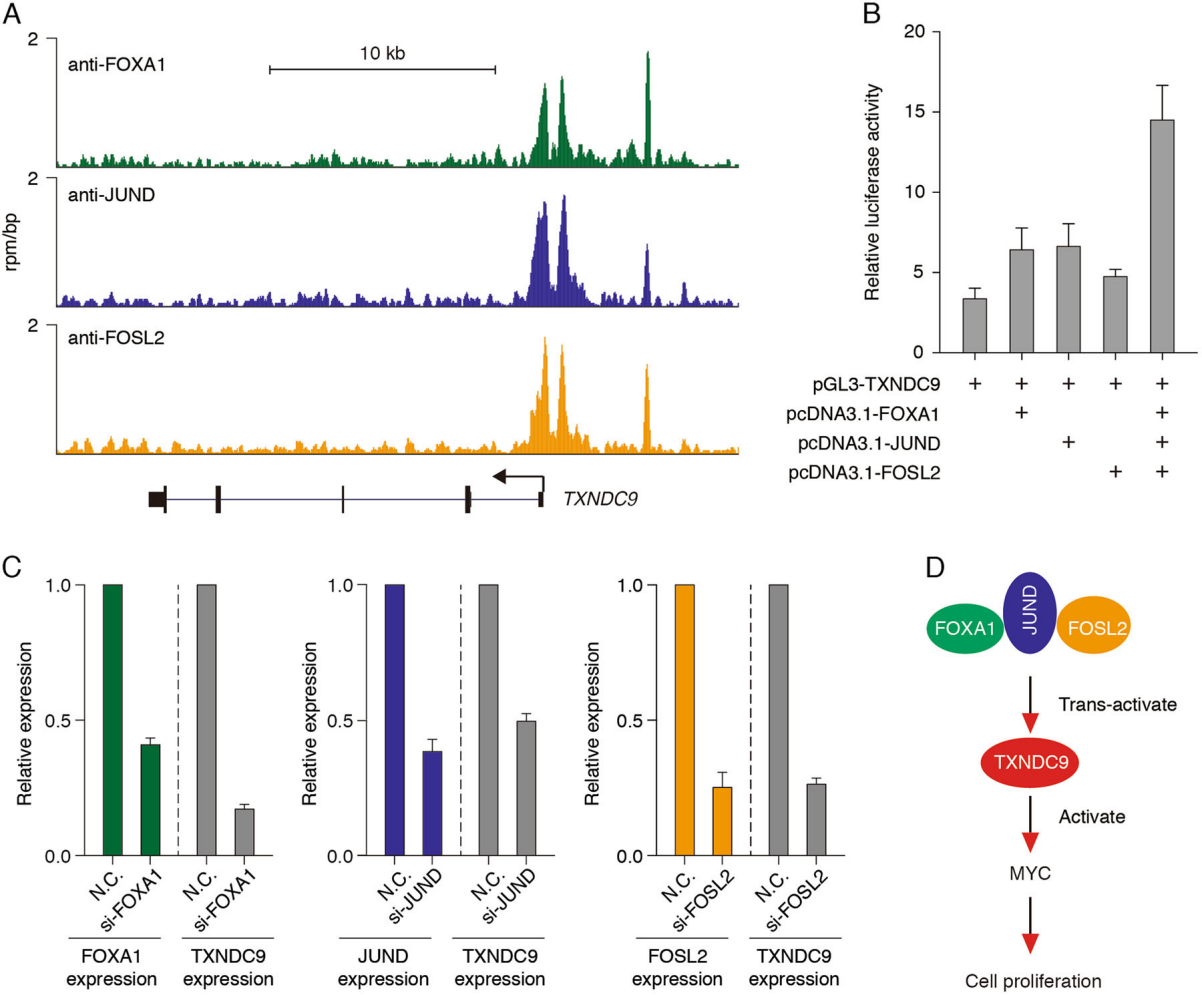
TXNDC9 was trans-activated by FOXA1, JUND, and FOSL2 in HepG2. **a** Overview of the loci of TXNDC9 in HepG2 cells. Refseq annotations are shown at the bottom. ChIP-seq datasets used in this analysis were retrieved from the NCBI Gene Expression Omnibus database. **b** FOXA1, JUND, and FOSL2 synergistically activated the promoter activity of TXNDC9. Dual luciferase reporter assays were used to evaluate the effects of FOXA1, JUND, and FOSL2 on the promoter activity of TXNDC9. Luciferase activity were normalized with cells transfected with pGL3-basic vector and pcDNA3.1 vector. **c** Knockdown FOXA1, JUND or FOSL2 decreased the expression of TXNDC9. Relative expression of TXNDC9 were detected in HepG2 cells transfected with FOXA1, JUND, FOSL2, or negative control (N.C.) siRNAs. Error bars represent the mean ± SD from three independent experiments. **d** Role of TXNDC9 in HCC. TXNDC9 was activated by FOXA1, JUND, and FOSL2 in HCC and overexpressed TXNDC9 promote the MYC-mediated transcriptional network and thus leads to cell proliferation of HCC

Next, we scanned the binding of transcription factors on the regulatory element of TXNDC9 in HepG2 cells. We found that FOXA1, JUND, and FOSL2 binding sites were significantly enriched at the promoter of TXNDC9, indicating a direct regulation of TXNDC9 by these transcription factors in HepG2 cells (Fig. 6a). To confirm the role of FOXA1, JUND and FOSL2 in TXNDC9 promoter regulation, we performed the luciferase activity assays using the promoter regions of TXNDC9, we found that FOXA1, JUND, and FOSL2 directly activated the promoter activity of TXNDC9 (Fig. 6b). Furthermore, we knocked down the expression of FOXA1, JUND, and FOSL2 in HepG2 with siRNAs. Knockdown of the expression of these transcription factors significantly decreased the expression of TXNDC9 in HepG2 cells (Fig. 6c). Hence, FOXA1, JUND, and FOSL2 directly drives TXNDC9 overexpression in HCC by transcriptional activation.

## Discussion

Thioredoxins have been reported to be involved in many malignant cancers, but their role in HCC remains unclear. In this study, we have demonstrated that TXNDC9, a member of thioredoxin superfamily, functioned as an oncogene in HCC. TXNDC9 was overexpressed in HCC and a high level of TXNDC9 was correlated with poor prognosis of HCC. Ablation of TXNDC9 significantly reduced the cancer phenotype of HCC. TXNDC9 directly interacted with MYC and stabilized the MYC protein and TXNDC9 knockout decreased the binding of MYC on chromatin. In addition, we demonstrated that TXNDC9 overexpression occurred via transcriptional activation by FOXA1, JUND, and FOSL2. Taken together, we have shown that the FOXA1/JUND /FOSL2-TXNDC9-MYC regulatory network promoted cancer progression of HCC (Fig. 6d).

In this study, we also demonstrated that TXNDC9 was over-expressed in HCC and associated with a larger tumor, poor histological type, and higher TNM stage. In addition, overexpressed TXNDC9 was associated with lower survival, suggesting that higher TXNDC9 expression could increase the progression and metastasis of HCC. Of note, another study has also observed overexpression in TXNDC9 in colorectal tumor tissues^16^. Although this study didn’t highlight the mechanism underlying TXNDC9 mediated oncogenesis, it emphasized the possibility of the important role of TXNDC9 in tumorigenesis. In addition, studies on TXNDC5, another member of the thioredoxin family, have revealed that it played a vital role in the proliferation and migration of tumor cells, acting as a tumor-enhancing gene^8,14,20^. Moreover, the tumorigenic effects of TXNDC5 have been described in cervical carcinoma^21^, lung cancer^22^, and liver cancer risk^23^. Taken altogether, the results from this and other studies demonstrate that thioredoxin overexpression might represent a signature in cancer, and that over-activated thioredoxin signaling might be a prognostic marker in cancer, and might also serve as potential therapeutic targets in cancer therapy.

In our study, we found TXNDC9 directly interacted with MYC and the knockout of TXNDC9 directly decreased the expression of MYC and its binding on chromatin, indicating that the importance of TXNDC9 in the protein levels of MYC and the transcription network governed by MYC. MYC is a key oncogene in gene regulation^24,25^. Amplification of MYC directly drives the tumorigenesis of many cancers by regulation of genes necessary for cell proliferation, cell invasion, and metastasis^26^. Studies on peroxiredoxins, another super-family of redox catalysts, demonstrated that Peroxiredoxin 1 promoted cell proliferation via direct interaction with MYC and alteration of the transcription of c-MYC targets^27^, suggesting a direct association between a redoxin domain containing protein and c-MYC. Generally, the thioredoxin super family is mostly involved in redox and oxidative progress regulation^27,28^. The thioredoxin domain of TXNDC9 could affect the oxidative stress and generate redox imbalance in HCC cells. In addition, many studies on MYC regulation have demonstrated a direct link between redox stress and MYC activation^29^. Thus, it may be that TXNDC9 overexpression directly alters the cellular redox state of HCC, and thus affects the transcriptional activity of MYC, which leads to HCC cell proliferation. Future studies need to be done to understand the association between thioredoxins and MYC function.

In addition to downstream factors involved in TXNDC9 mediated oncogenesis in HCC, we also observed that the upstream regulator of TXNDC9 was FOXA1, JUND, and FOSL2. We demonstrated that FOXA1, JUND, and FOSL2 co-localized at the promoter of TXNDC9. All three of these transcription factors contribute mainly to transcriptional activation in cell. Indeed, we observed that knockdown of the expression of these factors led to decreased expression of TXNDC9 in HepG2 cells. The Fos gene family members dimerize with the Jun family forming the transcription factor AP-1 complex, which trans-activates the transcription of its downstream targets^30^. Here we observed that the FOSL2, a member of Fos family, and JUND, a key member of Jun family, co-localized at the promoter of TXNDC9 and activated its transcription. FOXA1, JUND, and FOSL2 have previously been demonstrated to be involved in HCC^31,32^, but their association with thioredoxin have not been discussed. Our demonstration that FOXA1, JUND, and FOSL2 directly regulate TXNDC9 suggests that their role in tumorigenesis may be based on the regulation of thioredoxin domain containing proteins.

In conclusion, the current study demonstrated that the TXNDC9 was over-expressed in HCC and the overexpressed TXNDC9 was associated with poor prognosis of HCC. In addition, the FOXA1/JUND/FOSL2-TXNDC9-MYC axis is the basis of TXNDC9 mediated oncogenesis in HCC. Finally, the potential implication of TXNDC9 as a therapeutic target in HCC should be explored in future studies.

## Electronic supplementary material

Gene set enrichment analysis on biological process invovled in TXNDC9 knockout

Supplementary Figure Legends

TXNDC9 was overexpressed in HCC

Impact of TXNDC9 on the mRNA expression of MYC partners

MG132 induced the resorted expression of genes down-regulated upon TXNDC9 knockout/knockdown

Overexpression of MYC abolished the TXNDC9-knockout induced cell apoptosis

TXNDC9 localized in the cytoplasm in HCC cells

DNA methylation of TXNDC9 were similar and rare in HCC and adjacent normal tissues

Genes differentially expressed upon TXNDC9 knockout

Gene set enrichment analysis on molecular function invovled in TXNDC9 knockout
