# Supplementary material for: TXNDC9 promotes hepatocellular carcinoma progression by positive regulation of MYC-mediated transcriptional network
Source: Cell Death Dis. 2018 Oct 31;9(11):1110. doi: 10.1038/s41419-018-1150-4 (PMC6208382; doi:10.1038/s41419-018-1150-4)
Supplement: Supplementary file 2 — Supplementary Figure Legends [file 41419_2018_1150_MOESM2_ESM.docx]

**Supplementary Figure Legends**

**Figure S1.** TXNDC9 was overexpressed in HCC. TXNDC9 gene expression levels were assessed from GEO database. Error bars represent the mean ± SD.

**Figure S2.** Impact of TXNDC9 on the mRNA expression of MYC partners. TXNDC9 knockout induced modest regulation of MYC partners’ mRNA. mRNA expression of MXI1, MAX, MXD1 and MAZ in TXNDC9 knockout and wild-type HepG2 cells were detected by RNA-seq. Error bars represent the mean ± SD from three independent experiments.

**Figure S3.** MG132 induced the resorted expression of genes down-regulated upon TXNDC9 knockout/knockdown.

**Figure S4.** Overexpression of MYC abolished the TXNDC9-knockout induced cell apoptosis. Cell apoptosis were determined by the expression of Annexin V.

**Figure S5.** TXNDC9 localized in the cytoplasm in HCC cells. Cytoplasmic protein and nuclear protein were isolated and subject to western blot. The Lamin B was used as the marker of the nuclear protein, and the α-tubulin was used as the marker of the cytoplasmic protein.

**Figure S6.** DNA methylation of TXNDC9 were similar and rare in HCC and adjacent normal tissues. DNA methylation of TXNDC9 in HCC and adjacent normal tissues were retrieved from the HM450 DNA methylation Chip in TCGA database.
