# Supplementary figures and images for: TXNDC9 promotes hepatocellular carcinoma progression by positive regulation of MYC-mediated transcriptional network
Source: Cell Death Dis. 2018 Oct 31;9(11):1110. doi: 10.1038/s41419-018-1150-4 (PMC6208382; doi:10.1038/s41419-018-1150-4)

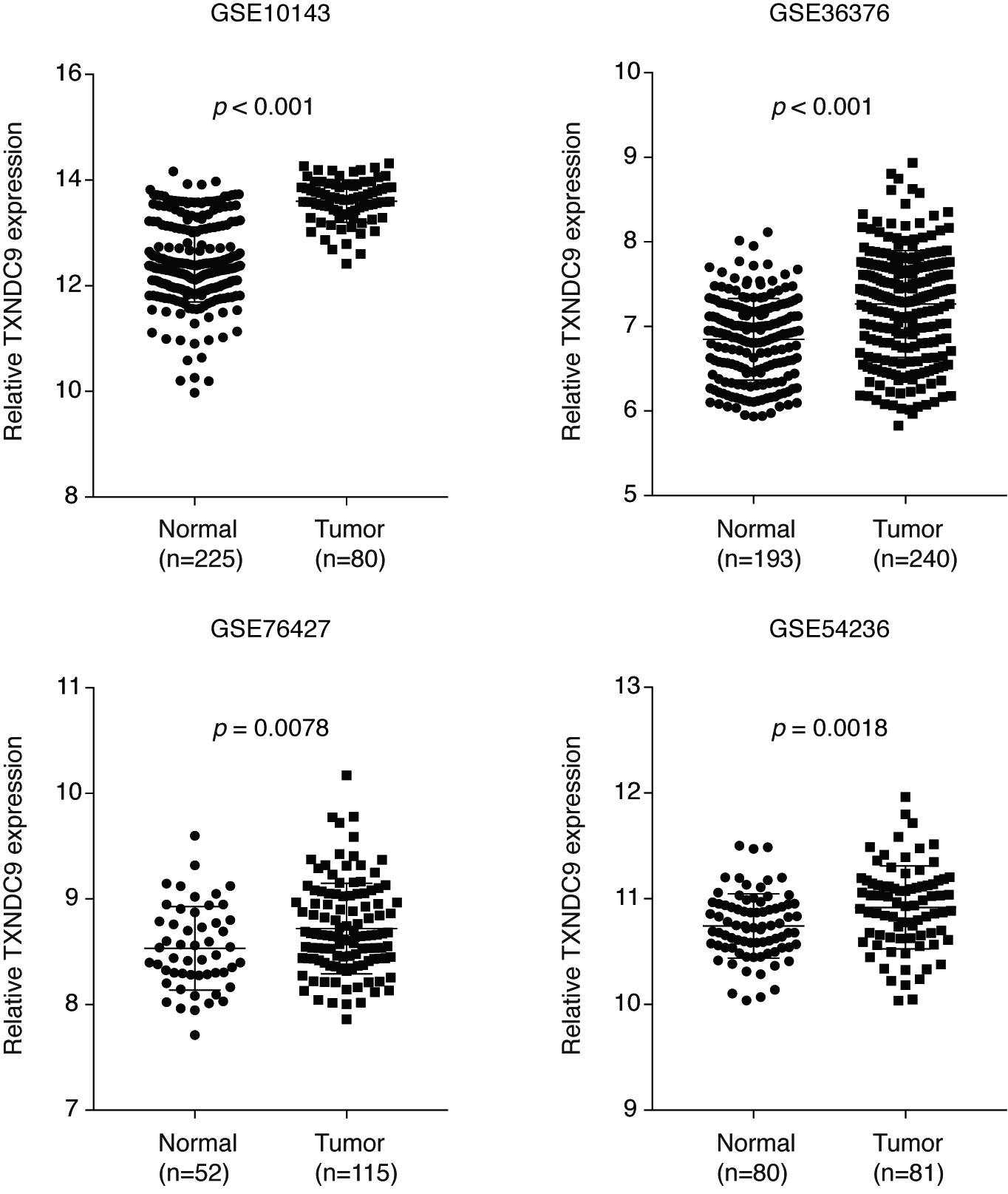

Supplement: Supplementary file 3 — TXNDC9 was overexpressed in HCC [file 41419_2018_1150_MOESM3_ESM.jpg]

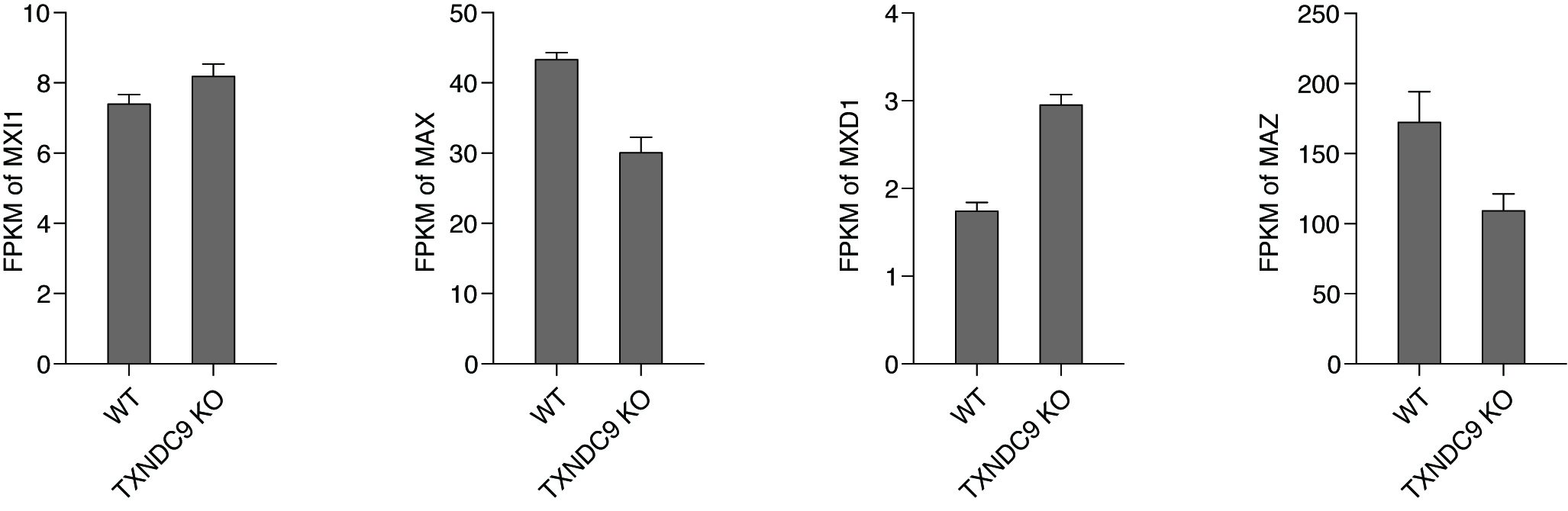

Supplement: Supplementary file 4 — Impact of TXNDC9 on the mRNA expression of MYC partners [file 41419_2018_1150_MOESM4_ESM.jpg]

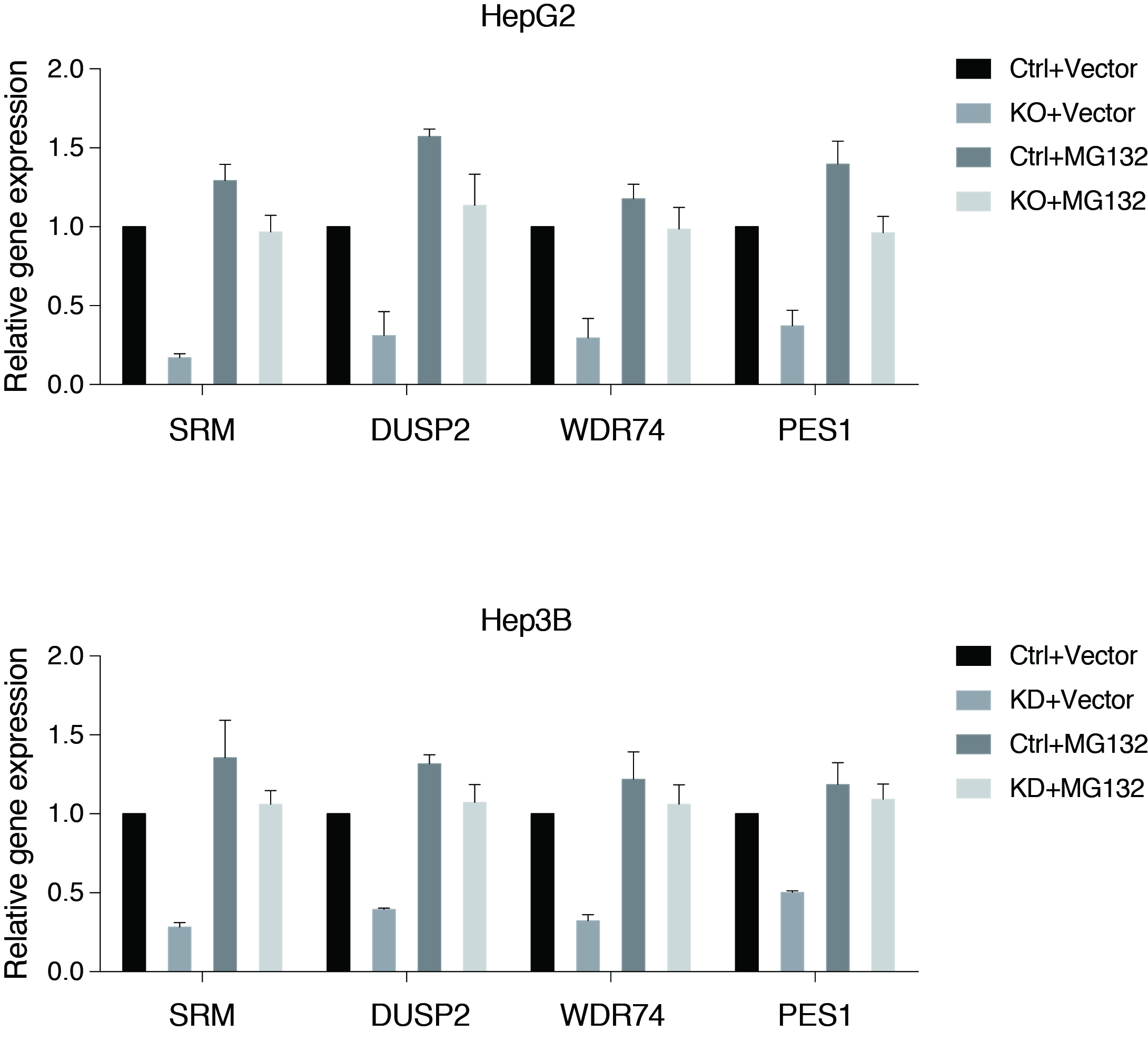

Supplement: Supplementary file 5 — MG132 induced the resorted expression of genes down-regulated upon TXNDC9 knockout/knockdown [file 41419_2018_1150_MOESM5_ESM.jpg]

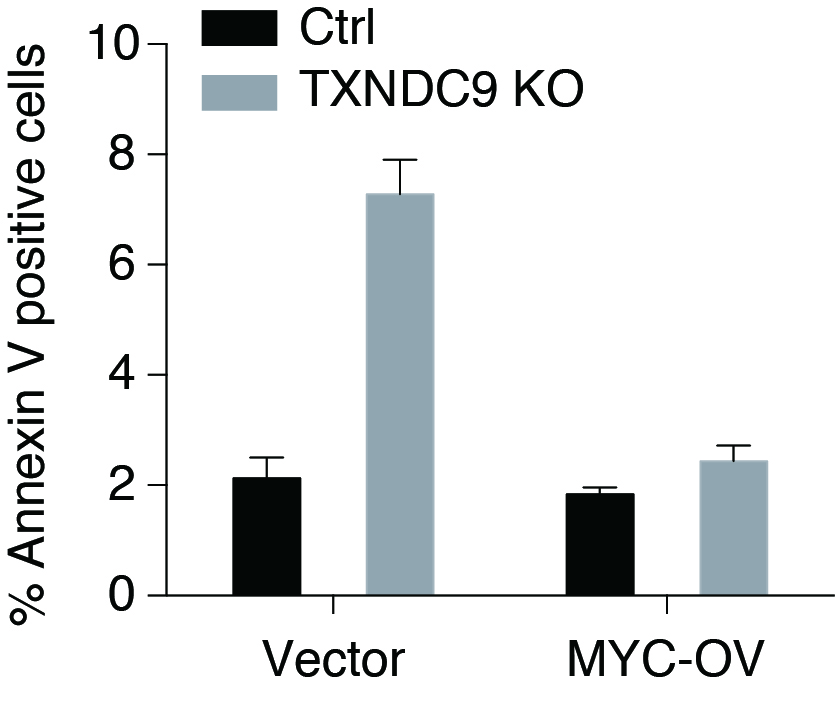

Supplement: Supplementary file 6 — Overexpression of MYC abolished the TXNDC9-knockout induced cell apoptosis [file 41419_2018_1150_MOESM6_ESM.jpg]

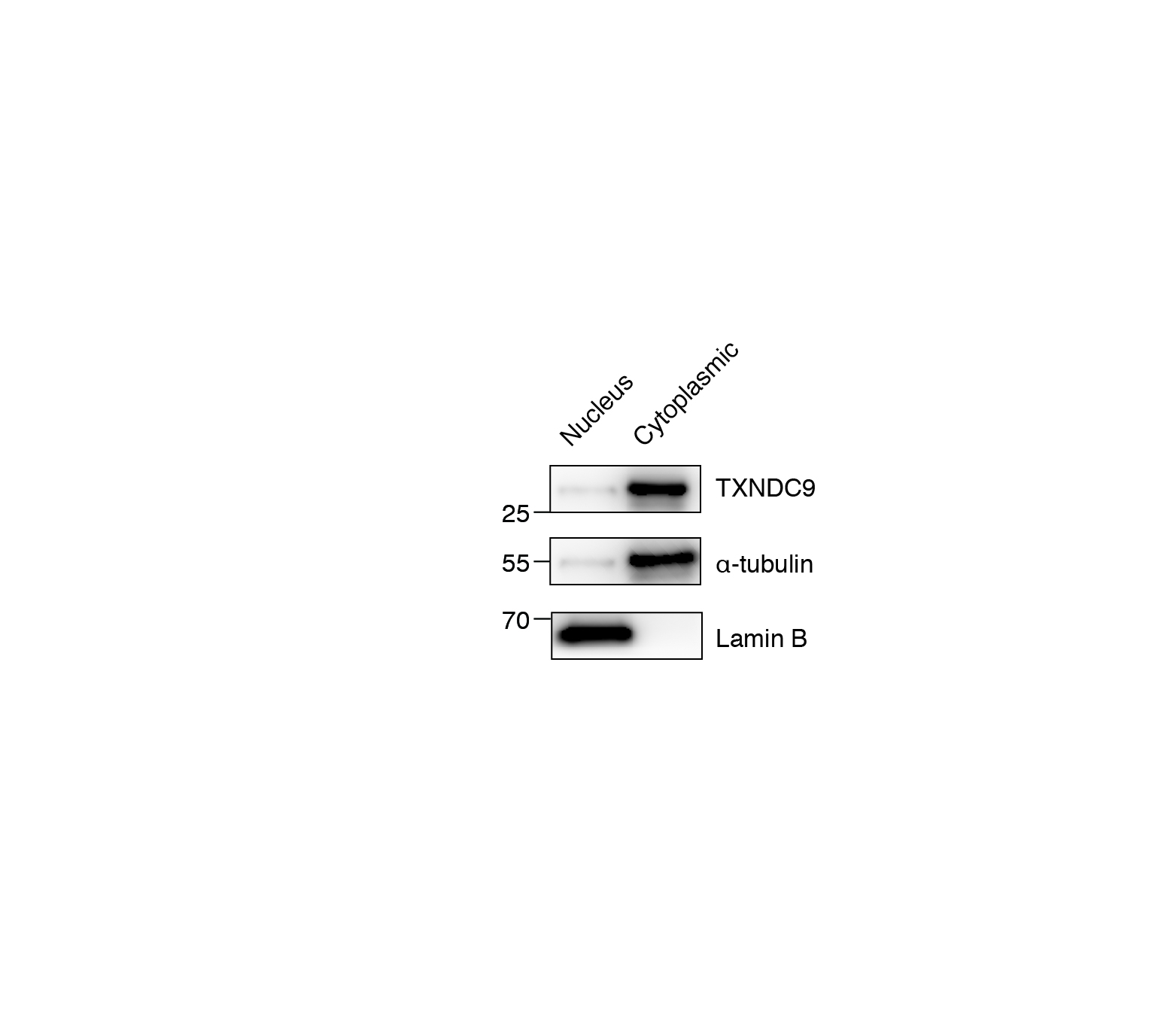

Supplement: Supplementary file 7 — TXNDC9 localized in the cytoplasm in HCC cells [file 41419_2018_1150_MOESM7_ESM.jpg]

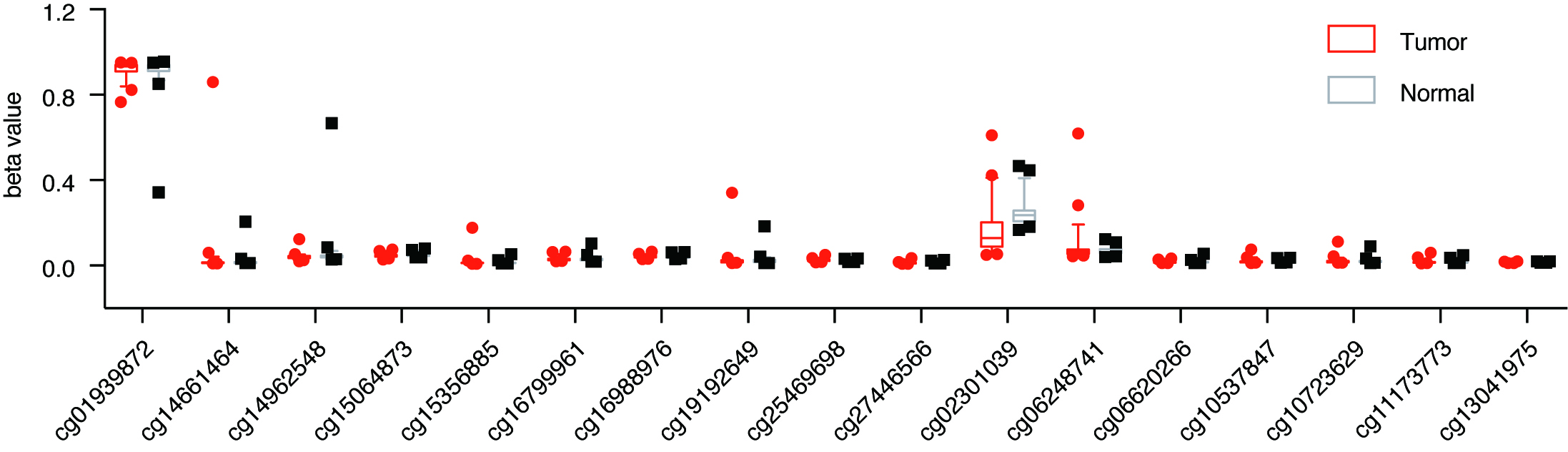

Supplement: Supplementary file 8 — DNA methylation of TXNDC9 were similar and rare in HCC and adjacent normal tissues [file 41419_2018_1150_MOESM8_ESM.jpg]
